# Privilege Tax of Dentists

**Published:** 1879-05

**Authors:** S. P. Cutler

**Affiliations:** Memphis, Tenn.


### ARTICLE VII.

### *Privilege Tax of Dentists in the Taxing District of Tennessee, the Phœnix of Old Memphis.*

BY S. P. CUTLER, M. D., D. D. S., MEMPHIS, TENN.

Our wise men of fame sent up to Nashville to make laws for the people, passed the following law, together with other privilege laws:

"All Chiropodists, Veterinary Surgeons or Dentists, $10 per annum."

The above is the order in which the bill reads. Barbers, somehow, were overlooked. They should have been included, as they once pulled teeth, bled, cupped and leeched.

Doctors and other specialtics of medicine, and lawyers are not taxed at all.

The category in which dentists are embraced, is certainly not very flattering to the pride of any accomplished dentist. Has it come to pass that dentists are to be classed with the other two professions by our wise men, our law givers? It is not presumed that a horse doctor or a corn trimmer is necessarily an educated man, though he may be such. I maintain that the tax is wrong and unjust in all the above named cases, more especially that of the dentists.

After the establishment of Dental Colleges and Associations all over the land, Journals filled with able scientific literature, to be ignored as professional men by our law makers is wrong, unjust, and contemptible in the extreme.

We are not as fortunate as any mechanic, as none of them are taxed. We are neither professional men nor mechanics in the eyes of the law. In the name of common sense what are we? Perhaps a middle man, perhaps something else; it is hard to tell what we are. We are taxed, notwithstanding, for the privilege of administering to the wants and sufferings of humanity, who seek relief daily and hourly at our hands.

